# The developmental origin of heart size and shape differences in *Astyanax mexicanus* populations

**DOI:** 10.1016/j.ydbio.2018.06.009

**Published:** 2018-09-15

**Authors:** Jocelyn L.Y. Tang, Yu Guo, William T. Stockdale, Kerisha Rana, Abigail C. Killen, Mathilda T.M. Mommersteeg, Yoshiyuki Yamamoto

**Affiliations:** a Department of Cell and Developmental Biology, University College London, London WC1E 6BT, United Kingdom; b University of Oxford, Department of Physiology, Anatomy and Genetics, South Parks Road, Oxford OX1 3PT, United Kingdom

**Keywords:** Heart development, Heart morphology, Organ size, *Astyanax mexicanus*

## Abstract

Regulation of heart size and shape is one of the least understood processes in developmental biology. We have for the first time analysed the hearts of *Astyanax mexicanus* and identified several differences in heart morphology between the surface (epigean morph) and cave-dwelling (troglomorph) morphs. Examination of the adult revealed that the troglomorph possesses a smaller heart with a rounder ventricle in comparison to the epigean morph. The size differences identified appear to arise early in development, as early as 24 h post-fertilisation (hpf), while shape differences begin to appear at 2 days post-fertilisation. The heart of the first-generation cross between the cave-dwelling and river-dwelling morph shows uncoupling of different phenotypes observed in the parental populations and indicates that the cardiac differences have become embedded in the genome during evolution. The differences in heart morphology are accompanied by functional changes between the two morphs, with the cave-dwelling morph exhibiting a slower heart rate than the river-dwelling morph. The identification of morphological and functional differences in the *A. mexicanus* heart could allow us to gain more insight into how such parameters are regulated during cardiac development, with potential relevance to cardiac pathologies in humans.

## Highlights

• Differences in heart size, shape and tissue structure between Astyanax populations.
• Furthermore, differences in cardiac melanophore and adipocyte numbers.
• Heart size and shape differences are apparent early in development.
• Surface and Pachón show differences in heart rate during development and adulthood.
• F1 hybrids show uncoupling of features observed in surface and Pachón populations.

## 1 Introduction

Tight control of heart morphology during development is crucial for normal cardiac function, as abnormalities can affect tolerance to varying physiological activities, ultimately affecting the overall viability of an organism. Many human congenital defects affecting heart size (hypertrophic cardiomyopathy), shape (single-ventricle defects) and tissue structure (non-compaction cardiomyopathy) are known to exist (Kloesel et al., 2016, Towbin et al., 2015), with genetic testing aiding in the identification of causative genes (Arndta and MacRae, 2014). Although the molecular and cellular biology of cardiovascular development has been intensely studied in a variety of vertebrates, the mechanisms behind the regulation of heart morphology is not clearly understood. Furthermore, it is not entirely known whether molecular mechanisms exist to confer cardiovascular differences to organisms with active or sedentary lifestyles. Organ size can be dictated by a wide variety of cellular activities, such as cell proliferation, apoptosis and fates of stem/progenitor cells and cellular hypertrophy. Several pathways have been implicated in heart size regulation, most notably the Hippo (Zhou et al., 2016) and FGF pathways (S. R. Marques et al., 2008).

Here, we introduce *Astyanax mexicanus* (Mexican cavefish) as a new model for understanding the regulation of heart size and shape. *A. mexicanus* is a single fish species comprising troglomorph (cave-dwelling) and epigean (surface) river populations. This species possesses unique traits that make it a useful model organism to study various aspects of adaptation to a food-scarce environment in perpetual darkness. Several cavefish populations are known to have derived from a surface fish ancestor, suggested from a couple of thousand to several million years ago (Fumey et al., 2016, Gross, 2012). Surface fish and cavefish are still inter-fertile and can produce fertile progeny, making genotyping studies such as a Quantitative Trait Locus (QTL) analysis feasible. Furthermore, due to its close ancestry to zebrafish, many tools are transferrable, including transgenesis and live imaging of embryos. By combining the unique traits of *A. mexicanus* and established tools in zebrafish, we can study how different selection pressures and genetic factors influence heart development.

Currently, the majority of teleost cardiovascular knowledge stems from zebrafish research. The adult fish heart is anatomically different from most vertebrates, with only a single atrium and ventricle. Blood from the body enters the atrium through the sinus venosus, is pumped to the ventricle, from where it is directed to the gills via the bulbus arteriosus. The blood is oxygenised in the gills, before directly being pumped towards the body (Simões et al., 2002). Cardiovascular development begins with the specification and differentiation of cardiac cell precursors in two bilateral heart fields, which migrate and fuse in the midline to form the cardiac disc, which gives rise to the initial linear heart tube. The heart tube then elongates by the addition of cells to its anterior and venous poles, and begins to beat by 24 h post fertilisation (hpf). By 48 hpf, the heart tube starts to loop and form chambers, acquiring an adult-like morphology by 5 dpf (Bakkers, 2011).

In this study, we provide the first characterisation of heart development, morphology and function in *A. mexicanus*. We show that while the hearts of both cavefish and surface fish have a general teleost fish morphology, there are several differences between the two. We find that shape and size differences of the heart arise early in development, suggesting that such traits are genetically determined. Moreover, heart rate measurements also reveal differences between the surface and cavefish. While functional differences appear to originate during developmental stages, coinciding with morphological differences, analysis of F1 hybrids indicates uncoupling of phenotypes.

## 2 Materials and methods

### 2.1 Ethical approval and fish husbandry

The experiments were performed on laboratory stock of teleost *Astyanax mexicanus*, surface fish and Pachón, Tinaja and Chica cavefish. All the experimental procedures were performed in accordance with the UK Animals (Scientific Procedures) Act 1986 and institutional guidelines. Laboratory strain of surface fish were originally collected at Nacimiento Del Rio Choi, San Luis Potosí, Mexico. The three laboratory strains of cavefish population are originally from Cueva de El Pachón in Tamaulipas, Mexico (Pachón cavefish), El Sótano de la Tinaja (Tinaja cavefish) in San Luis Potosi, Mexico, and La Cueva Chica (Chica cavefish) originally provided by the Steinhart Aquarium (San Francisco, CA USA). The fish used in this study have been bred for multiple generations at the Yamamoto laboratory after having been obtained from University of Maryland (Jeffery laboratory). All the fish were maintained and fed once a day at 20 °C in pH 7.2 tank water with a 14-h light and 10-h-dark light cycle. Embryos were obtained by temperature induced spawning after increasing the tank water temperature to 25 °C.

### 2.2 Embryo collection and fixation

Embryos were collected and incubated in petri dishes at 25 °C in zebrafish embryo medium 2 (15 mM NaCl, 0.5 mM KCl, 0.05 mM Na_2_HPO_4_, 0.15 mM KH_2_PO_4_, 1 mM CaCl_2_, 1 mM MgSO_4_·7H_2_O, 0.7 mM NaHCO_3_). At specific time stages, embryos were fixed in 4% paraformaldehyde in PBS overnight at 4 °C. Embryos were then dehydrated gradually by increasing gradients of methanol/PBS and stored at − 20 °C.

### 2.3 Adult heart isolation

Fish hearts of surface and Pachón cavefish were isolated at 50-days, 6-months, 1 year and 2.5-years old and hearts of Tinaja and Chica cavefish were isolated at 6-months old. The fish were anaesthetised and killed using MS222 (Tricaine methanesulfonate, Sigma, A5040, 0.16 mg/ml), after which the standard body length from snout to tail base and the body weight were measured. Then the fish were placed on a cut sponge in a ventral-side-up orientation. Spring scissors were used to make an incision to penetrate the thorax and open the pericardial sac. The ventricle was exposed and hearts were isolated by cutting the artery superior to the bulbus arteriosus and the veins inferior to the atrium. The isolated hearts were directly fixed in 4% paraformaldehyde in PBS at 4 °C overnight, followed by washing with PBS and stored in PBS containing 0.05% sodium azide at 4 °C or processed for paraffin embedding.

### 2.4 Paraffin embedding and sectioning

To visualise the transparent embryos in paraffin, the embryos were transferred from 100% methanol to 0.1% eosin staining in 100% ethanol to stain the embryos for 30 s, followed by 3 washes with 100% ethanol. The ethanol solution was replaced by 1-butanol, the samples transferred to liquid paraffin wax and kept at 65 °C for 30 min. After washing twice with liquid paraffin wax, embryos were embedded. For embedding of adult hearts, after 4% paraformaldehyde fixation, the hearts were dehydrated by increasing ethanol gradients (70%, 80%, 90%, 95%, 100% twice, 1 h per step), kept in 1-butanol overnight, after which the hearts were transferred to liquid paraffin wax at 65 °C. After washing twice for 1 h with liquid paraffin wax, the hearts were embedded. 7–12 µm sections were cut using a microtome (AO Spencer 820) and mounted onto Superfrost slides (Thermo Scientific), then dried overnight at 37 °C.

### 2.5 Acid Fuchsin Orange-G (AFOG) staining and fluorescent immunohistochemistry

Sections were deparaffinised by xylene and rehydrated by decreasing ethanol gradients (100% three times, 95%, 90%, 80%, 70%, 50%) to PBS, or distilled water in case of AFOG staining. For AFOG staining, the sections were placed in Bouin’s fixative at 60 °C for at least 2 h and washed in distilled water until sections were clear. The sections were then placed in aqueous 1% phosphomolybdic acid for 5 min, again rinsed in distilled water, before stained with AFOG solution for 5 min (5 g/l Water Blue, 10 g/l Orange G, 15 g/l Acid Fuchsin (Sigma), pH1.09). The sections were quickly rinsed in distilled water before dehydration and mounting using DPX (Sigma).

For fluorescence immunohistochemistry, 30 min of 3% (w/w) hydrogen peroxide was followed by high-pressure antigen retrieval with antigen unmasking solution (H-3300, Vector Laboratories Inc.). Sections were then blocked for 30 min with TNB (0.1 M Tris-HCl, pH = 7.5: 0.15 M NaCl; 0.5% blocking powder). The primary antibody against monoclonal mouse anti-myosin heavy chain 2 (MF20) (1:50 in TNB, Developmental Studies Hybridoma Bank) was incubated with samples overnight at room temperature. AlexaFluor 488 goat anti-mouse IgG secondary antibody (1:200 in TNB) and DAPI (2.5 μg/ml; Sigma) were incubated for 2 h at room temperature. Immunohistological stained sections were mounted with hydromounting solution (Applied Genetic Technologies Corporation) or Mowiol 4-88 (Applichem).

### 2.6 Whole mount immunohistochemistry and light sheet imaging

2 dpf embryos were rehydrated from 100% methanol to PBS followed by 10 min of proteinase K treatment at 65 °C (10 µg/ml in PBS). After washing in PBS-T, the embryos were fixed in 4% paraformaldehyde for 10 min and blocked for 2 h in TNB followed by primary antibody (MF20) overnight at room temperature. After washing in PBS-T, the embryos were incubated with secondary antibody and DAPI (2.5 μg/ml; Sigma) for 2 h (AlexaFluor 488 goat anti-mouse IgG secondary antibody, 1:200 in TNB). After washing in PBS-T, the embryos were embedded in 0.5% agarose/PBS in glass capillaries for light sheet microscopy (Zeiss Z1).

### 2.7 3D reconstructions and volume measurements

Pictures of stained sections were taken by fluorescent microscope (Leica DM4500 LED). Three-dimensional (3D) reconstructions of hearts were performed using Amira (Visage Imaging). Pictures of sections were aligned automatically, followed by manual adjustment. Light sheet data was loaded directly. Different regions of the heart were labelled by the brush tool with the MF20 or AFOG staining as a reference. The 3D reconstructions were made by this programme according to the labelling.

### 2.8 Heart rate measurement

Embryos were selected at the specific stages and anaesthetised with 0.16 mg/ml M MS-222 for 5 min, in separate 6-well culture plates. The working MS-222 solution was made by diluting the stock 4 mg/ml MS-222 with E2 medium (for 3 dpf and 5 pf embryos) or in tank water (for 10 dpf embryos). The embryos were then moved to 24-well culture plates and incubated at 25 °C for 25 min. Following incubation, the embryos (that had been immersed in MS-222 for approximately 30 min) were recorded under an inverted microscope (×25 magnification, Sanyo, Xacti VPC-FH1 Full HD) for 30 s.

For adult heart rate measurements, Pachón were anaesthetised in 0.16 mg/ml MS222 for 20 min, while surface and F1 generation fish were anaesthetised for 10 min. This difference was needed as Pachón fish were not fully anaesthetised until 20 min, whereas surface fish and F1 took 10 min. Additional experiments with few Pachón at 10 min and surface fish and F1 at 20 min only enhanced observed differences (Surface fish 20 min vs Pachón 20 min, surface = 46.5, Pachón = 23.9, p = 0.0022). When fully anaesthetised, the fish were placed ventral side up in a cut sponge and the heart rate was filmed for 1 min (Sanyo, Xacti VPC-FH1 Full HD). The heart beat was visible through the body wall and did not require opening of the thorax.

### 2.9 Cloning heart development genes and RNA probe synthesis

RNA from 5 dpf, 6 dpf surface and 24 h Pachón cave fish was extracted using TRIzol Reagent (Thermo Fisher) following the RNA isolation TRI Reagent protocol (Sigma-Aldrich). cDNA was synthesised according using RT-PCR. The probes were made using the following primers: *myh6* forward TTTCTGCGTTCTGAAAGGGC, reverse GAAACAGCGTGGCTTTGACA, *myl7* forward CAAGCTGACTGGTGCCATTAT and reverse Reverse GAAGAGCCCTTCTTCTTTCCA. PCR fragments were purified using QIAquick Purification Kit (Qiagen). The blunt PCR fragments were subject to A-tailing with GoTaq Flexi DNA polymerase and cloned into pGEM-T vector (Promega) and RNA probes were generated using DIG RNA Labelling Kit (Roche).

### 2.10 Whole mount in situ hybridisation

In situ hybridisation was performed as described (Thisse and Thisse, 2008), with several modifications. The hybridisation mix was made with 65% formamide and the incubation temperature for pre-hybridisation and subsequent washes was set at 65 °C. Once the stain has developed, the colouration reaction was stopped by 3 washes in PBST (PBS with 0.1% Tween-20) followed by 4% paraformaldehyde in PBS at 4 °C overnight. Embryos were then dehydrated through a successive series of methanol and stored at − 20 °C overnight before imaging by microscopy.

### 2.11 Microscopy and image analysis

Brightfield images were taken with the Nikon Digital Sight stereo microscope (SMZ1500, Nikon) using the NIS Elements F 2.30 software. Measurements of the adult hearts were done using Nikon ATC-2U software. A triangle surrounding the ventricle in lateral view was drawn onto the images of each heart and the blank area of the triangle, which was not covered by the ventricle, was measured to analyse ventricle shape. Measurements as shown in the main figures were corrected for body length. As an additional correction factor we used bulbus arteriosus area, which gave similar results to body length (Supplementary Fig. 1). In addition, the number of pigmented melanophores and the number of adipocytes attached to the epicardium of hearts were counted as visible. Especially in surface fish, adipocytes were present in large clusters of overlying cells, which may have resulted in an underestimation of cells. Measurements of the 3D-reconstructed hearts and embryos were done similar to the adult hearts using Fiji (ImageJ) software.

### 2.12 Statistics

To test for statistical significance of measurement differences, one-way ANOVA, Dunnett’s Multiple Comparison Test or independent 2-tailed, Student’s T-test for unpaired samples was used, as indicated in the figure legends. P < 0.05 is denoted by *, P < 0.01 is denoted by **, P < 0.001 by *** and< 0.0001 by ****.

## 3 Results

### 3.1 Heart morphology in *Astyanax mexicanus*

**Fig. 1:**
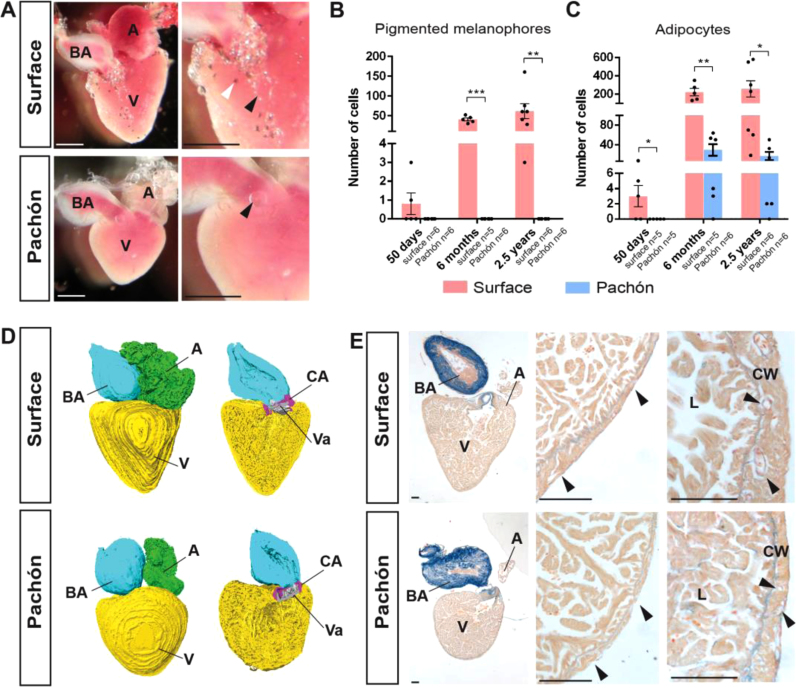
Morphology of the *Astyanax mexicanus* heart. (A) Comparison of the adult surface and Pachón heart, showing the atrium (A), ventricle (V) and bulbus arteriosus (BA). Arrowheads point to melanophores (white) and adipocytes (black). Quantification of (B) melanophores and (C) adipocytes in both morphs at 50 days, 6 months and 2.5 years (significance shown for Student’s *t*-test. P < 0.05 is denoted by *, P < 0.01 is denoted by **, P < 0.001 by ***). (D) 3D-reconstructions of the heart showing the exterior and interior structures such as the conus arteriosus (CA, purple) and valves (Va, grey). (E) AFOG stained heart sections of both morphs showing the variable thickness of the compact wall in both fish, but with a generally thinner wall in Pachón. AFOG stains collagen in blue and myocardium in orange. Surface fish have a large number of coronary vessels in the compact wall (arrowheads), Pachón have these in the compact wall where the wall is thicker (L: lumen; CW, compact wall; scale bars 100 µm).

Comparison of the outside appearance of the adult Pachón and surface fish heart immediately draws the eye to differences in cell populations present on the heart. Most surface fish hearts have clearly visible pigment cells on the surface of the heart. These melanophores are most abundant on the bulbus arteriosus and on the basal side of the ventricle, with a larger number of cells present in older fish. In line with its global body albinism, the Pachón heart lacks these pigmented melanophores at any of the stages examined (Fig. 1A–B). In addition to the difference in pigment cells, there is a strong difference in cardiac fat cell population between the two fish. Surface fish have a large amount of adipocytes present on the surface of the heart. Similar to the melanophores, these cells start to appear around 50 dpf and increase in numbers with age. They are mainly present on the basal side of the ventricle, between the ventricle and the atrium, and between the ventricle and bulbus arteriosus. While cavefish are known to have a much higher level of body adipocytes (Aspiras et al., 2015, Hüppop, 2000, Jeffery, 2009), there are only few adipocytes visible on their hearts. Interestingly, these few adipocytes are much larger than the cells found on the surface fish heart (Fig. 1A, C). To further analyse the hearts, we made three-dimensional reconstructions. These reconstructions show that, similar to the zebrafish, both the Pachón and surface fish heart are ‘modern teleost’ hearts. These ‘modern teleost’ hearts can be distinguished from ‘ancient’ teleost hearts by a better developed ventricle with a myocardial conus arteriosus, which is separated from the bulbus arteriosus by a single row of outflow tract valves (Grimes and Kirby, 2009). Both Pachón and surface fish hearts have a myocardial conus arteriosus with a single row of valves (Fig. 1D. conus arteriosus in purple, valves in grey). Additionally, ‘modern’ teleost hearts can be divided into 4 different categories, type I–IV, classified by the structure of the ventricular myo-architecture. The type I hearts have a spongy ventricular myocardium without compact layer, while type II–IV hearts have trabeculae surrounded by a compact wall with limited (type II) to extensive (type IV) coronary circulation (Grimes and Kirby, 2009). Like zebrafish, *A. mexicanus* hearts fall within the type II category. Both Pachón and surface fish have a compact wall with coronary vessels present within this wall, especially surface fish have clearly visible intramural coronary vessels (Fig. 1E, arrowheads). Wall thickness is variable in both fish, but overall thicker in surface fish. Intramural coronary vessels in Pachón are less abundant, and are mainly present in the areas where the wall is thicker (Fig. 1E). In neither fish coronary vessels are observed within the trabecular area.

### 3.2 Identification of the morphological differences in the adult heart

**Fig. 2:**
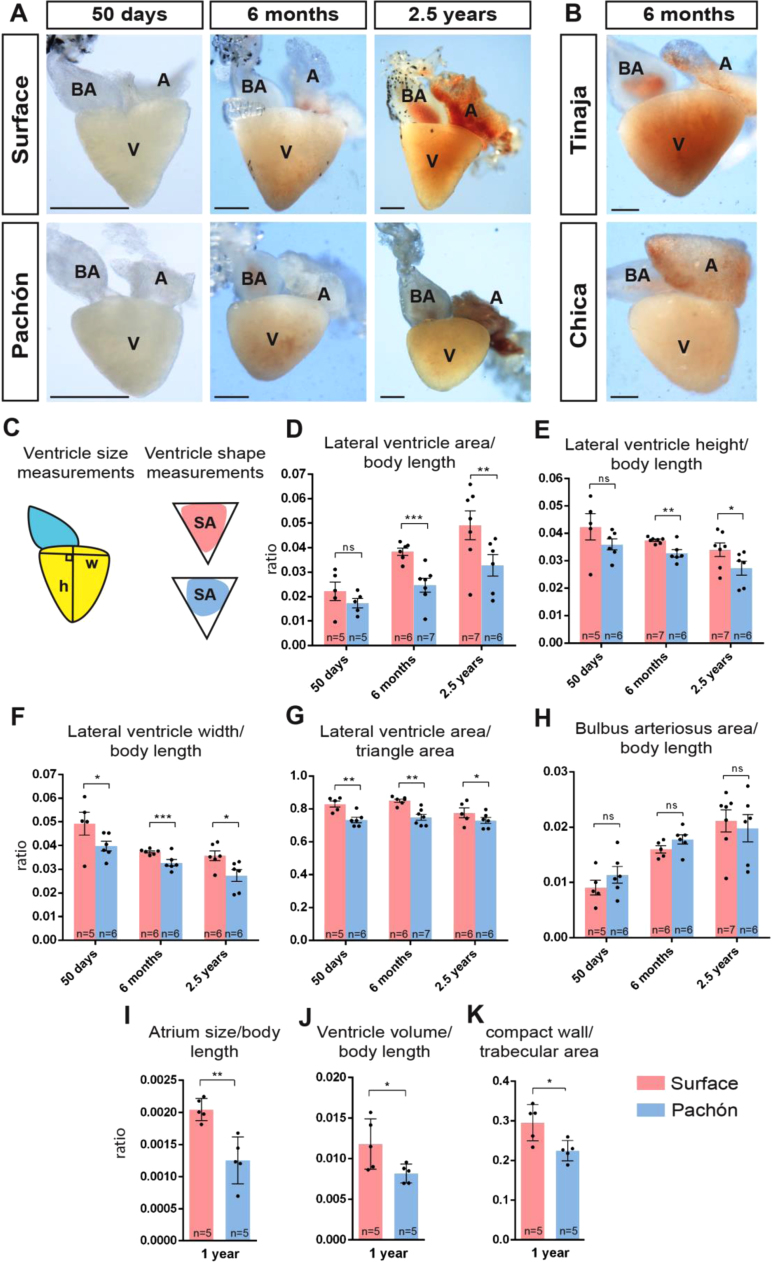
Comparison and quantification of the adult ventricle. (A) The adult heart in lateral view at 50 days, 6 months and 2.5 years of age. (B) The adult heart of Tinaja and Chica cavefish populations at 6 months of age. (A: atrium, V: ventricle, BA: bulbus arteriosus, scale bars: 50 µm) (C) Parameters determined for measuring the ventricle area (yellow) height (h), width (w), bulbus arteriosus area (blue) and shape (SA, surface area). Graphs showing measurements for (D) ventricular area, (E) ventricle height, (F) ventricle width, (G) ventricle shape and (H) bulbus arteriosus area, all standardised by body length. Graphs comparing (I) atrium size, (J) ventricle volume and (K) tissue composition of the heart at 1 year of age. The lower the compact wall/trabecula area ratio the spongier the heart (significance shown for Student’s *t*-test. P < 0.05 is denoted by *, P < 0.01 is denoted by **, P < 0.001 by ***, and ns means not significant).

**Fig. 3:**
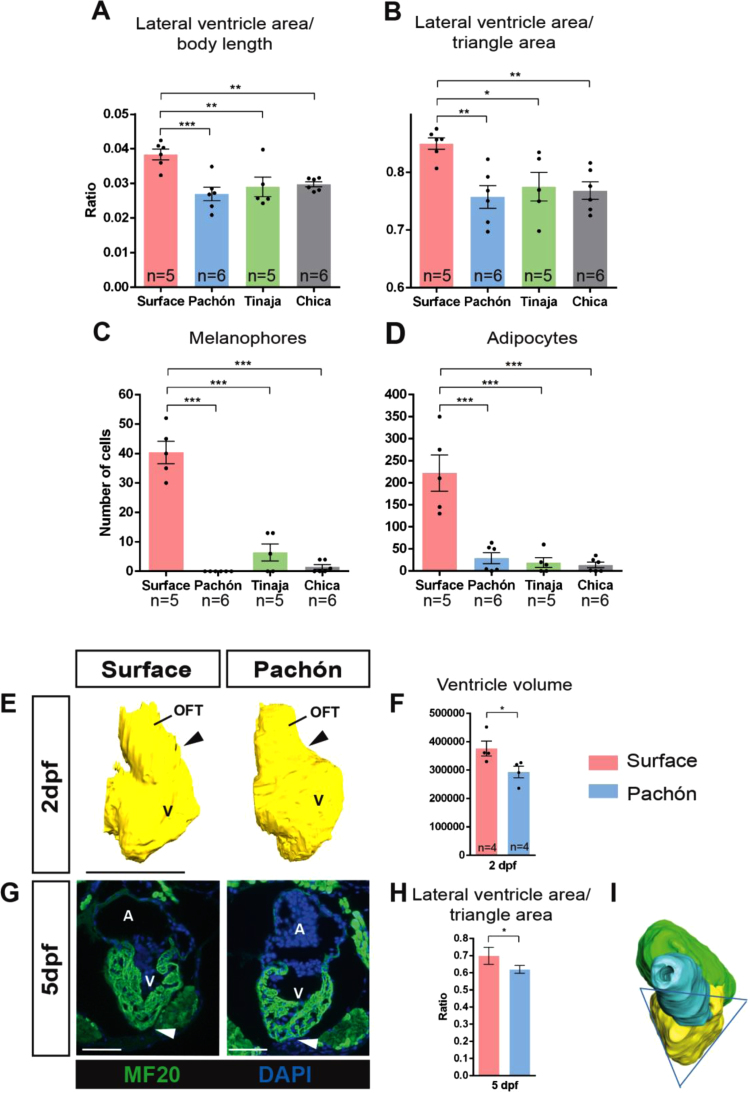
Comparison of (A) lateral ventricle area (p_ANOVA_ = 0.0012), (B) shape (p_ANOVA_ = 0.0048), (C) melanophore count (p_ANOVA_ ≤ 0.0001) and (D) adipocyte count (p_ANOVA_ ≤ 0.0001) of surface fish and the three cavefish populations at 6 months of age (significance shown for Dunnett’s multiple comparisons test). (E) 3D reconstructed ventricles at 2 dpf. Arrowheads point to morphological differences already visible at 2 dpf, with the ventricle showing a bigger bend towards the outflow tract in Pachón compared to surface fish (white arrow). (F) Graph comparing ventricular volumes at 2 dpf and (significance shown for Student’s *t*-test). (G) Immunostained sections at 5 dpf. Arrowheads point to the rounder ventricle visible at 5 dpf. (H) Graph comparing roundness of the ventricle at 5 dpf (significance shown for Student’s *t*-test). (I) 3D-reconstruction of heart at 5 dpf showing measurement (triangle) for shape analysis. P < 0.05 is denoted by *, P < 0.01 is denoted by **, P < 0.001 by ***. A: atrium, V: Ventricle, OFT: outflow tract, scale bars: 100 µm.

While the overall structure of the hearts seems very comparable, the shape and size of especially the ventricles appeared different, with the ventricle of the surface morph appearing larger and more triangular (particularly at the apex) than its Pachón counterpart. In order to characterise the morphology of the adult heart of the epigean morph and other cave populations of *A. mexicanus* in detail, hearts were dissected from fish at 3 different ages: 50 days, 6 months and 2.5 years. As shown in Fig. 2A, morphological differences in the ventricle are already visible at 50 days and persist into later adulthood. Examination of other cavefish populations such as Chica and Tinaja (Fig. 2B) also display the round ventricle morphology at 6 months of age. To quantify the size differences observed, the height, width and surface area of the ventricle in lateral view were measured and standardised by whole body length (Fig. 2C). The surface area and height of the epigean morph ventricle is significantly larger than the Pachón troglomorph at 6 months, whereas differences in the width of the ventricle can be observed from 50 days (Fig. 2D–F). Body length, which was used for correction, was similar between the fish measured (Supplementary Fig. 1A). The differences in shape were measured based on the area that the ventricle occupied within an equilateral triangle drawn to touch all three sides of the ventricle (Fig. 2C). From 50 days onwards, the Pachón fish possess a rounder ventricle than the surface morph (Fig. 2G). Aside from the ventricle, we also quantified the lateral surface area of the bulbus arteriosus, a thick-walled structure connecting the ventricle and the aorta (which functions to maintain continuous blood flow to the gill arches). However, no differences were observed between the 2 morphs (Fig. 2H). As the adult ventricle is smaller in Pachón compared to surface fish, we next wondered whether the atrium was also smaller in the troglomorphs. In contrast to the thicker walled ventricle that keeps it shape after isolation, the thin atrial wall collapses without blood flow and is therefore more difficult to measure. As an alternative approach, through 3D-reconstruction of sectioned hearts, we quantified the volume of the atrial as well as ventricular myocardium of 1 year old fish. The Pachón heart has both a smaller atrial and ventricular volume in comparison to the epigean morph (Fig. 2I–J). As a rounder ventricle has been linked to a spongier heart, we next compared how spongy the ventricles are by measuring the ratio of compact wall volume vs trabecular volume. Indeed, the Pachón ventricle is not only rounder, but also spongier than that of the surface fish (Fig. 2K). Measurement of the various dimensions and shape of the ventricle in other cavefish populations such as Tinaja and Chica show a similar phenotype to the Pachón (Fig. 3A–B), with no significant differences observed in the bulbus arteriosus when compared to the epigean morph (data not shown). Similarly, Tinaja and Chica populations also show little to no melanophores or adipocytes on the heart surface (Fig. 3C–D). In contrast to Pachon, Tinaja and Chica cavefish have a low number of melanophores present on the heart (Fig. 3C). This is consistent with the fact that fish from these caves are not completely albino and have some pigment cells on their body (Jeffery, 2009).

### 3.3 Size and shape differences during embryonic development

Once the morphological differences in the adult heart were established, our next aim was to determine if these differences are already present during development. Embryos stained for myocardial marker MF20 already show differences in morphology at 2 dpf, when the heart is still merely a linear tube, with the ventricle showing a larger bend in the outer curvature towards the outflow tract (Fig. 3E). Additionally, volume measurements indicate that the ventricle of the surface fish is larger than the of the Pachón at this early stage of development (Fig. 3F). At 5 dpf, the atrium and ventricle have ballooned out from the initial heart tube and the bulbus arteriosus has developed, producing a morphology similar to that observed in the adult heart (Fig. 3G–I). As the heart is still very small at this stage, to be able to measure the heart, we generated 3D reconstructions of sections stained for myocardium, which were then analysed for roundness. Interestingly, the ventricle shape in the Pachón morph already appears to be rounder than the surface morph at 5 dpf (Fig. 3H–I).

**Fig. 4:**
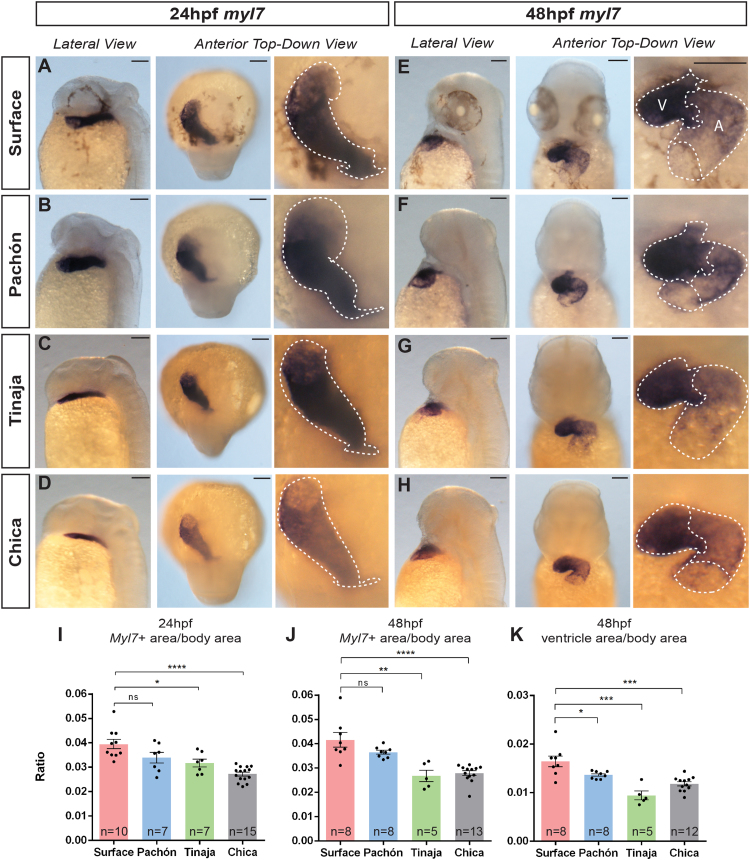
*myl7* expression in *A. mexicanus* during early development. Brightfield images showing expression pattern of *myl7* at (A–D) 24 hpf and (E–H) 48 hpf for surface and cavefish populations (A: atrium, V: ventricle). Dotted lines show outline of expression pattern, indicating the tube structure (at 24 hpf) and differential expression in the ventricle at 48 hpf. (Scale bars: 100 µm) Comparison of whole expression area at (I) 24 hpf (p_ANOVA_ ≤ 0.0001) and (J) 48 hpf (p_ANOVA_ ≤ 0.0001) and (K) just the ventricle at 48 hpf (p_ANOVA_ ≤ 0.0001) between *A. mexicanus* populations (significance shown for Dunnett’s multiple comparisons test. P < 0.05 is denoted by *, P < 0.01 is denoted by **, P < 0.001 by *** and< 0.0001 by ****, ns means not significant).

Next, we visualised the hearts at earlier stages of development through assessing the expression of heart-specific genes. We cloned *A. mexicanus* cardiac muscle gene myosin light chain 7 (*myl7*) and examined its expression pattern by in situ hybridisation. In all the *A. mexicanus* populations examined, *myl7* is uniformly expressed in the heart tube at 24 hpf but at 48 hpf, its expression is more concentrated in the ventricular than the atrial region (Fig. 4A–H). This expression pattern is similar to that seen in zebrafish (Pi-Roig et al., 2014). *myl7* expression at 48 hpf also reveals slightly differing heart tube morphologies between the surface and cave morphs. Measurements of the whole expression area in dorsal view (after standardisation by whole body area in side view) revealed significant differences between the surface and cave populations except for the Pachón at 24 hpf (Fig. 4A–D, I). This trend is again observed at 48 hpf when the entire *myl7* expression area is taken into account (Fig. 4E–H, J). However, when only the ventricular area is measured at 48 hpf, the epigean morph is significantly larger than all troglomorph populations observed, consistent with the volume measurements in Pachón (Figs. 3F, 4K).

**Fig. 5:**
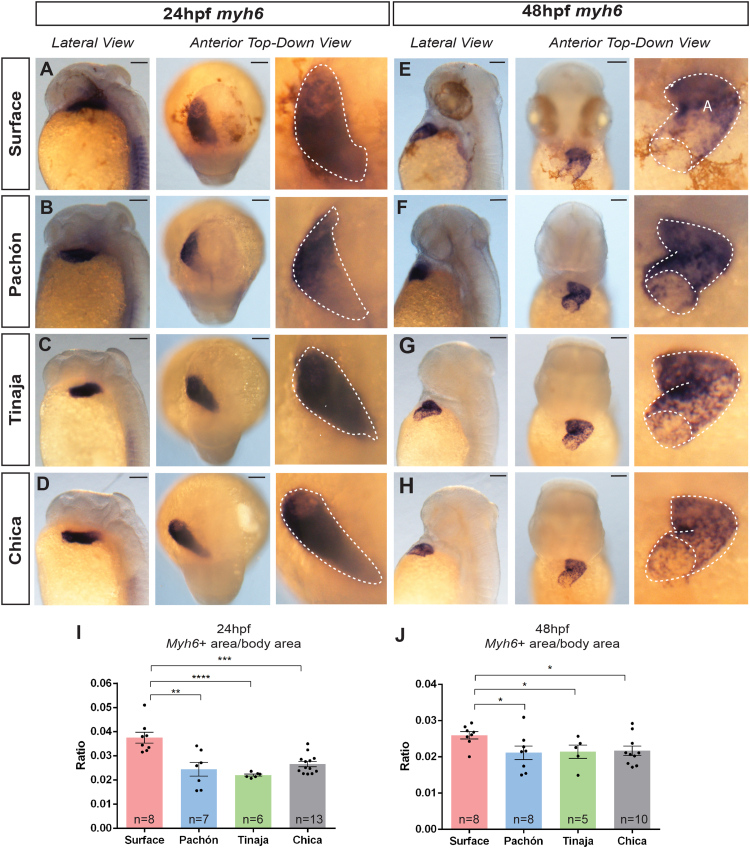
*myh6* expression in *A. mexicanus* during early development. Brightfield images showing expression pattern of *myh6* at (A–D) 24 hpf and (E–H) 48 hpf for surface and cavefish populations (A: atrium). Dotted lines show outline of expression pattern, indicating the tube structure at 24 and 48 hpf. (Scale bars: 100 µm) Comparison of expression area at (I) 24 hpf (p_ANOVA_ ≤ 0.0001) and (J) 48 hpf (p_ANOVA_ = 0.0963) between *A. mexicanus* populations (significance shown for Dunnett’s multiple comparisons test. P < 0.05 is denoted by *, P < 0.01 is denoted by **, P < 0.001 by *** and< 0.0001 by ****).

The volume and area of the *A. mexicanus* ventricle is larger in the surface morph from at least 24 hpf for Tinaja and Chica populations. By 48 hpf, all cavefish populations observed had a smaller ventricular area than the epigean morph, with shape differences detectable as early as 5 dpf. To investigate the atrium during early cardiac development, we cloned myosin heavy chain 6 (*myh6*), a gene that is specific for atrial myocardium in zebrafish (Singh et al., 2016). At 24 hpf, *myh6* is expressed in a sub-region of the heart tube in both the epigean morph and troglomorphs (Fig. 5A–D). At 48 hpf, the tubular structure of the atrium can be seen from the expression pattern of *myh6* (Fig. 5E–H). The atrial tube appears to be shorter and more curved in the cavefish populations than the epigean morph. Quantitative analysis of the *myh6* expression shows that the surface morph has a larger area than the all troglomorph populations examined. While the area of myh6 expression is smaller in cavefish, the level of *myh6* expression seems increased. These differences are detectable from 24 hpf and are maintained at 48 hpf (Fig. 5I–J).

### 3.4 Heart morphology of surface and Pachón F1 hybrids

**Fig. 6:**
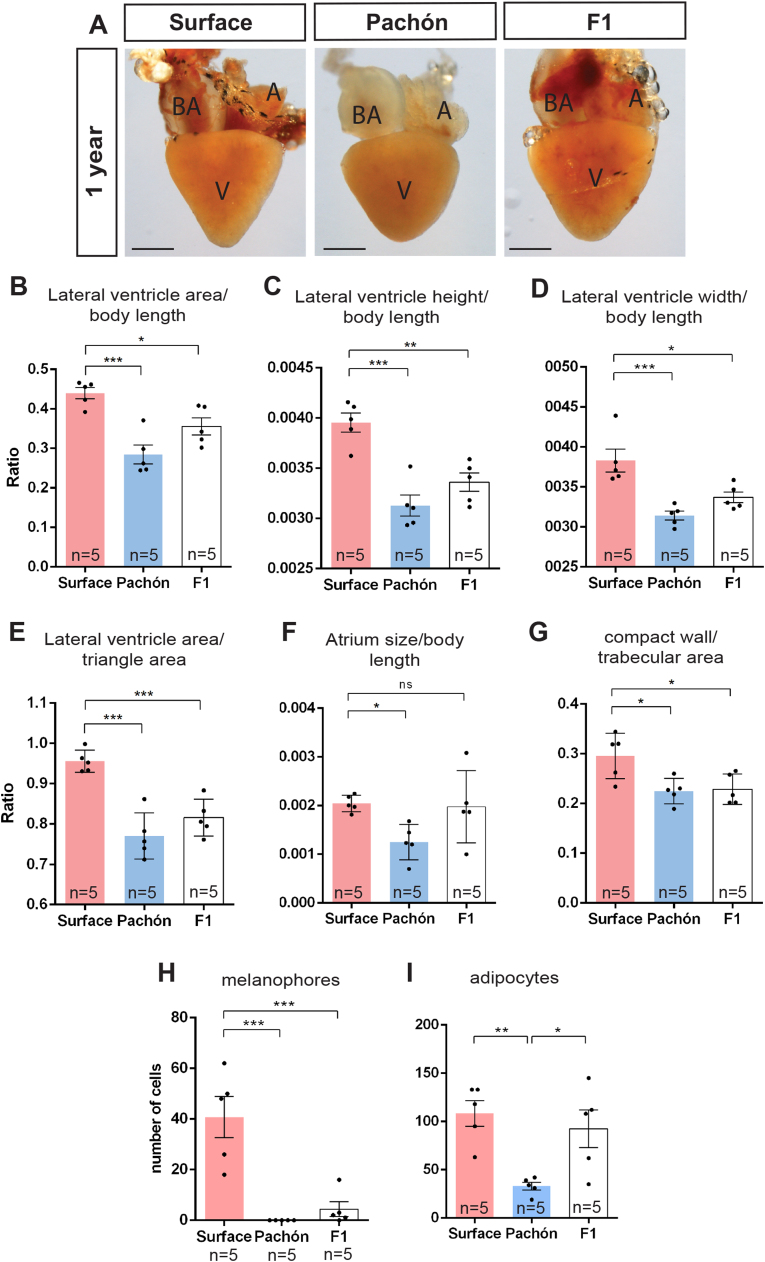
(A) Images of the adult heart at 1 year of age for surface, Pachón and F1 hybrid generation fish. (A: atrium, V: ventricle, BA: bulbus arteriosus; scale bars: 50 µm). Graphs showing (B) ventricle area, (C) ventricle height), (D) ventricle width, (E) shape, (F) atrium size and (G) tissue composition of the heart at 1 year of age. The lower the compact wall/trabecula area ratio the spongier the heart (significance shown for Dunnett’s multiple comparisons test. P < 0.05 is denoted by *, P < 0.01 is denoted by **, P < 0.001 by *** and ns means not significant).

As our data suggested that the shape and size differences of the heart have a developmental origin, we hypothesised that such traits are genetically heritable. We examined the adult heart of the F1 hybrid generation of surface and Pachón fish at 1 year of age. Interestingly, the measurement of ventricle area, width and height showed that F1 hybrid fish have a ventricle size more similar to Pachón than surface fish (Fig. 6A–D). The shape of the ventricle appears to be rounder in both the F1 and Pachón fish when compared to the surface morph (Fig. 6E). The ventricular volume however, does not seem to differ between the surface, Pachón and F1 fish in the hearts that were quantified (data not shown). No differences were detected when the area of the bulbus arteriosus was measured in all 3 populations, a result which is also observed at other time points (data not shown). Interestingly, in contrast to the ventricle measurements, quantification of atrial size suggests that the F1 fish have a similar volume to the surface morph (Fig. 6F). Myocardial tissue of F1 hybrid hearts also appear to be less spongy than surface fish and more similar to the Pachón cavefish (Fig. 6G). Melanophores are present on the F1 hearts, but in very low numbers, while in contrast, adipocytes numbers are more similar to the surface fish (Fig. 6H–I). These data indicate that the cardiac differences between the cavefish and surface fish are indeed genetically determined. Additionally, they show uncoupling of phenotypes, with atrial size and adipocyte number similar to surface fish and ventricular size, shape and sponginess similar to Pachón.

### 3.5 Functional differences between the epigean and troglomorphic heart

**Fig. 7:**
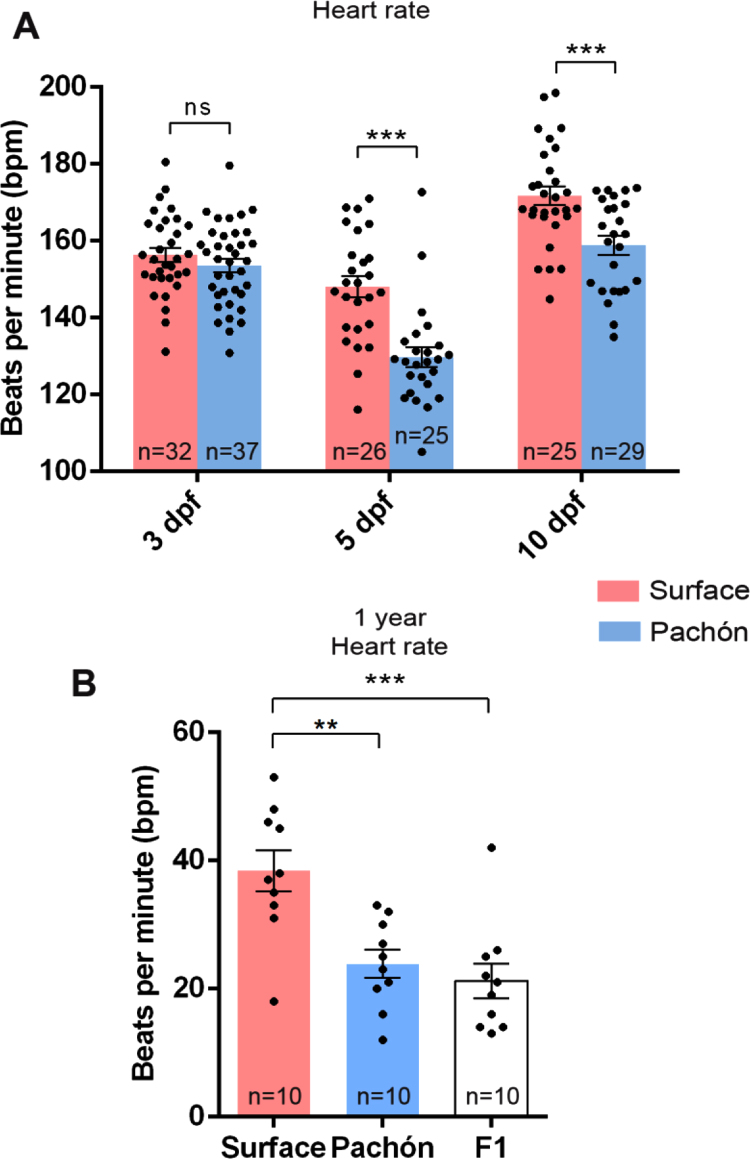
(A) Heart rate at different developmental time points for the surface and Pachón fish populations (significance shown for Student’s *t*-test). (B) Heart rate at 1 year of age for the surface, Pachón and F1 hybrid generation. (p_ANOVA_ = 0.0136; significance shown for Dunnett’s multiple comparisons test. P < 0.01 is denoted by **, P < 0.001 by *** and ns means not significant).

After the characterisation of numerous morphological differences of the heart of the surface fish, cavefish and the F1 hybrid, we postulated whether this correlated to any functional differences. We therefore measured the heart rate of anesthetised surface and Pachón fish in vivo. At early developmental stages, the heart rate of *A. mexicanus* is higher than in the adult stages and significantly different heart rates are observed from 5 dpf onwards (Fig. 7A). At 5 and 10 dpf, surface fish have a higher heart rate than Pachón. Additionally, we also compared the heart rate of the F1 hybrid generation to both the surface and Pachón morphs at 1 year of age. At this adult stage, the heart rate slows down significantly in comparison to developmental stages but the surface fish maintain a higher rate than the Pachón fish. Interestingly, the F1 hybrid fish exhibit a heart rate similar to Pachón (Fig. 7B).

## 4 Discussion

*Astyanax mexicanus* has been a valuable research model for different research fields for decades, however, its heart has been ignored so far. Here, we have identified differences in heart size, morphology, melanophore and adipocyte cell populations, and beating frequency. These differences already occur very early in development, but become increasingly apparent over life. The ventricle of the Pachón fish heart is not only smaller than the surface fish ventricle, it is also spongier and much less triangular in shape. The same holds true for the other cavefish populations examined. As these differences already occur very early during heart development, before external factors start to play a role, this suggests that the differences in morphology are genetically regulated. Moreover, the heart of the first-generation cross between the cave-dwelling and river-dwelling morph displays a mixture of phenotypes similar to either of the two parental populations, further indicating that the genetic determinants are quantitatively inherited. Morphological differences partially uncouple with functional differences. As such, *A. mexicanus* could be a valuable new model for identifying the molecular and cellular mechanisms underlying heart cell populations, and size and shape regulation.

## 5 The Pachón heart has few adipocytes and no pigmented melanophores

While the Pachón heart does not have pigmented melanophores, these cells can be found in varying degrees on surface fish hearts. It is not clear yet why there are pigment cells present on the heart, as the organ is located within the body and thus not exposed to sunlight. The presence of melanophores on the heart is not unique to *Astyanax mexicanus*. This has also been observed in the mouse and human heart (Brito and Kos, 2008, Mjaatvedt et al., 2005), though they have not been reported to be present on the epicardial surface of the ventricle. Previous studies have shown that the development and migration of neural crest cells and melanophore precursors is normal in cavefish populations, however, there is a reduction in the number of melanophores and decreased melanin production (Jeffery, 2009, McCauley et al., 2004). Pachón melanophores still have the ability to convert L-tyrosine into melanin in the melanosome, but the transportation of L-tyrosine from cytoplasm to the melanosome is blocked (Jeffery, 2009). It is not clear yet if the Pachón heart has colourless melanophores.

In addition, surface fish have a large amount of fat cells on the surface of the heart, mainly located in the atrioventricular groove and between the bulbus arteriosus and ventricle. It is interesting that surface fish store less adipose cells in the body but have more adipocytes on the heart and vice versa in cavefish (Aspiras et al., 2015, Hüppop, 2000, Jeffery, 2009). The function of adipocytes on the fish heart has not yet been investigated and if this difference in adipocyte population is related to the other differences observed between the fish is unknown. The large epicardial fat deposits on the human heart have been shown to be metabolically active and produce a number of factors that modulate cardiac structure and function, immune control, angiogenesis and control of proliferation, but that are also associated with the development of heart disease, e.g. coronary artery disease (Hassan et al., 2012). They might have similar roles in the fish heart. While ventricular morphology and heart rate of the F1 hybrids is similar to Pachón, the number of fat cells is similar to surface fish, indicating this is a dominant surface fish feature.

## 6 The surface fish heart has a more triangular shape, while the Pachón heart has a spongier structure

While both the Pachón and surface fish hearts are comparable to the zebrafish heart and follow the general pattern of a ‘modern teleost’ type II heart (Grimes and Kirby, 2009), there are clear differences between the hearts of the two morphs. First of all, while both morphs can be considered to have a triangular shaped ventricle, the ventricle of the surface fish has a more pointed triangular shape than that of Pachón and the other cavefish populations examined. In teleosts, there have been several observations of pyramidal hearts correlating with active lifestyles, while rounder hearts are found in species that are relatively inactive (Santer and Greer Walker, 1980, Santer et al., 1983, Tota et al., 1983). Therefore, the larger and more triangular heart in surface fish compared to Pachón suggests adaptation to a more active life in the rivers compared to the caves. A more triangular shaped heart is suggested to correlate to a less spongy heart. This indeed seems to be the case in *A. mexicanus*. Surface fish that have a more triangular shaped heart, also have a less spongy heart, which means a larger ratio of compact wall to trabeculae. A thicker outer compact layer of myocardium has been shown to be an adaptation required by fish which use large amounts of metabolic energy for activities such as long migrations, constant fast swimming against a strong current or hunting. For example, teleost flatfish like the Plaice have a spongy rounder heart, while highly migratory teleost Salmon have a triangular shaped heart (Santer and Greer Walker, 1980). A heart with more trabeculae has a larger surface area exposed to the blood and as a result likely has a higher capacity for oxygen uptake. This is beneficial during high activity, but is also considered the reason that fish living in low oxygen environments, such as deep-sea fish, have a larger amount of trabeculae, allowing adequate gas exchange between the myocardium and the circulating oxygen-low blood (Santer and Greer Walker, 1980). As water in the caves has been shown to be hypoxic (Hüppop, 1986), a spongier heart would be a useful adaption to cave life. Interestingly, sedentary teleosts such as the icefish have a spongy heart with no compact myocardium and pump larger volumes of a blood at a slow rate (Tota and Gattuso, 1996), in contrast to fast-swimming tuna which have a compact myocardium generating higher blood pressures accompanied by a higher heart rate (Agnisola and Tota, 1994). The fact that the Pachón ventricle is also smaller might again relate to a less active life style. However, in this study, we have only analysed lab reared fish; the cavefish are kept in the fish facility under the same conditions as surface fish. This suggests that during the adaptation to hypoxic, still water in caves, these differences have become permanently embedded in the genome. This is further supported by the fact that the first differences between the morphs can already be identified at 24 hpf, when both fish are still merely inactive and do not feed yet. Both morphs show synchronous development when kept under the same temperature and dissolved oxygen, including simultaneous start of cardiac contractions at 21 hpf (Hinaux et al., 2011). We confirmed this, as well as analysed the development of the otoliths in the vestibular labyrinth, at 26 and 50 hpf, which again confirmed synchronous development between surface fish and cavefish (Supplementary Fig. 2). This indicates that the observed differences are not likely the result of differences in growth speed between the surface fish and cavefish. Additionally, the heart morphology and function of the F1 cross between Pachón and surface fish is surprisingly similar to Pachón, while the swimming activity resembles the surface fish, possibly due to the acquisition of vision (Kowalko et al., 2013).

## 7 The differences in heart size and morphology are genetically regulated

In addition to the factors mentioned above, exercise and hypoxia, also differences in temperature, food deprivation and light cycle have been shown to influence heart size and beating frequency (Gamperl, 2004; I. J. Marques et al., 2008). The cavefish inhabit a perpetually dark environment where food is scarce and temperatures fluctuate much less than in the rivers (Hüppop, 1987). All these factors have very likely exerted certain selection pressures on the heart of the troglomorph that the epigean morph did not have to face. Energy conservation is an important factor for survival in situations where food is limited. Certain adaptations in cavefish appear to be linked to energy conservation, such as the elimination of eyes and metabolic circadian rhythm (Moran et al., 2015, Moran et al., 2014). The heart is an energetically costly organ, due to its constant requirement for contraction (Kolwicz et al., 2013). Therefore, a reduction in its size and beating frequency would be an effective method of saving energy, especially for organisms with relatively sedentary lifestyles. While our data shows that the cardiac differences between the fish are genetically determined, we cannot exclude other factors external to the heart that may also play a role. The early differences in heart size and shape can be secondary to differences in physiology between surface fish and cavefish. For example, it is not known if there is a difference in blood pressure between the two fish, but a higher blood pressure and peripheral resistance in blood vessels can affect the development of the heart. The resistance measured in zebrafish embryos at 37 hpf is very low, but not negligible (Hove et al., 2003).

The developmental processes that lead to a change in heart size are likely due to the regulation of cell specification, proliferation and apoptosis, or a combination of these mechanisms. The Hippo signalling pathway is known to negatively regulate cell proliferation, survival and to some extent cell growth (Stanger, 2008). Hippo signalling has been investigated in zebrafish in the context of regeneration but in general this pathway is not very well understood in vertebrates (Morrisey, 2011). However, an observation in the mouse suggests that reduced Hippo signalling leads to a larger heart size due to increased cell proliferation. A reduction in Hippo signalling also promotes Wnt signalling, a pathway involved in cardiomyocyte proliferation and differentiation (Heallen et al., 2011). Alternatively, heart size may be regulated by varying the number of cardiac cell progenitors early in development. In zebrafish, overexpression of the heart-specific transcription factor Nkx2.5 leads to a proportionally expanded heart (Chen and Fishman, 1996). Reduced retinoic acid signalling is also known to reduce the number of cardiomyocytes (Keegan et al., 2005), while Nodal and Wnt signalling (Brand, 2003) are involved in myocardial progenitor specification, all being suitable candidates to changing heart size.

The reduced size of both the atrium and ventricle in cavefish during early heart development suggests decreased contribution of second heart field progenitors to both the arterial and venous poles of the heart tube. While the initial heart tube is derived from the first heart field, the cells added to the heart after initial tube formation are called the second heart field progenitors (Bakkers, 2011). The sinus node as well as the atrioventricular node are added to the venous pole of the heart during second heart field addition. The sinus node is the primary pacemaker of the heart which controls heart rate. Therefore, the smaller atrium suggests that also sinus node development might be affected in cavefish. This is supported by the higher levels of atrial *myh6* expression in cavefish, which suggest further advanced myocardial differentiation compared to the surface fish, as less new diluting progenitor cells are added. If sinus node development is altered in cavefish, this would be a likely explanation of heart rate reduction.

Most cardiomyocytes in the fish heart not only remain mononucleated throughout life, they also retain the capacity for proliferation (Uygur and Lee, 2016). This capacity allows the adult fish heart to repair itself after injury, in contrast to the human heart. In human, after birth, the majority of cardiac growth is obtained through hypertrophy. Although some cardiomyocytes retain the ability to proliferate over life, a large majority of cardiomyocytes become multinucleated and polyploid, while losing their ability for proliferation (Uygur and Lee, 2016). *A. mexicanus* will be a valuable model to understand how hearts size control is regulated in the mononucleated proliferating fish heart. The zebrafish has been a popular model organism within the fields of cardiovascular development and heart regeneration. Our identification of the morphological and functional differences in the *A.mexicanus* heart demonstrates its potential as a new model system for studying cardiovascular development and pathologies. Further genetic and molecular characterisation of the phenotypes shown may enable us to elucidate the mechanisms behind organ shape and size, as well as their relationship with function. Moreover, large-scale drug testing that has been done with zebrafish (Kithcart and MacRae, 2017) may also be possible with *A. mexicanus*, with potential relevance to human cardiac defects and disease.
