# Supplementary material for: The developmental origin of heart size and shape differences in Astyanax mexicanus populations
Source: Dev Biol. 2018 Sep 15;441(2):272–84. doi: 10.1016/j.ydbio.2018.06.009 (PMC6142174; doi:10.1016/j.ydbio.2018.06.009)
Supplement: Supplementary file 1 — Supplementary material [file mmc1.pdf]

Supplementary Figures

Supplementary Figure 1

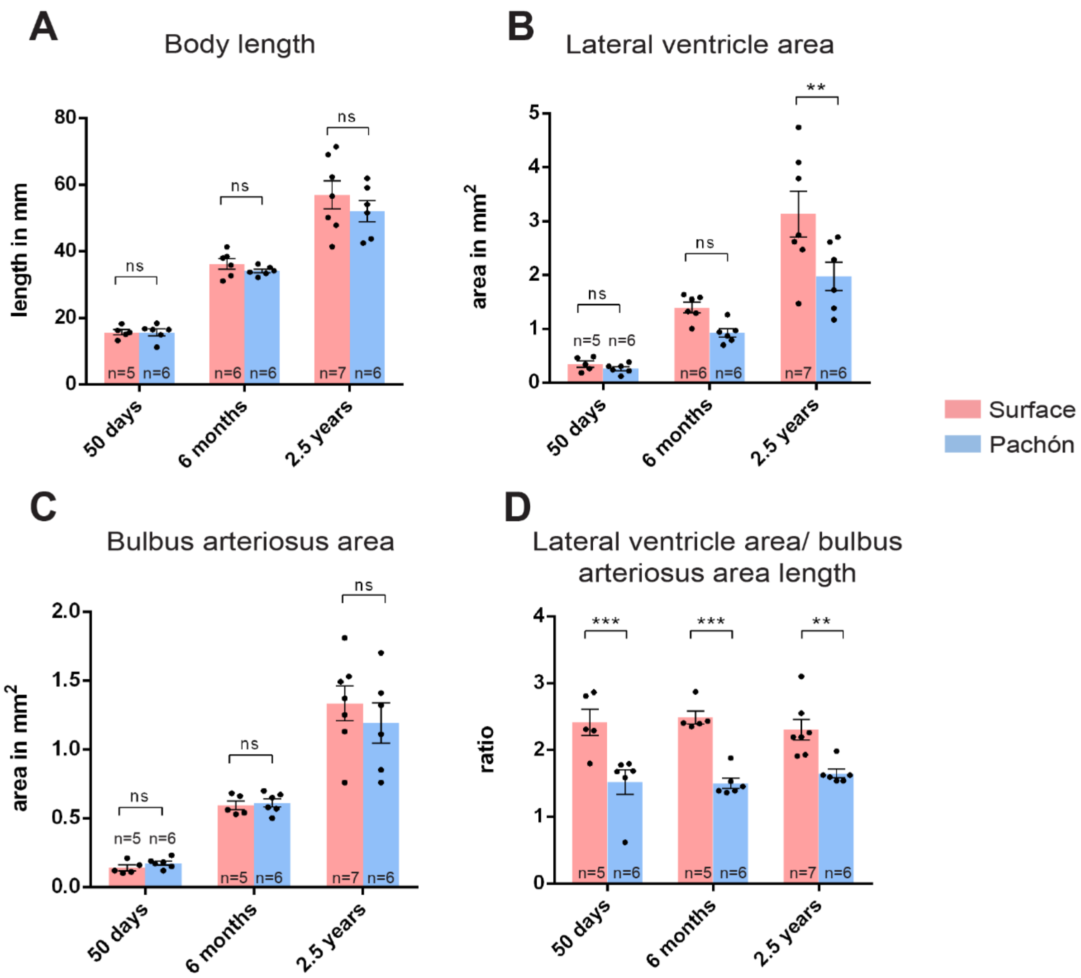

Supplementary Figure 1. (A) No differences in body length between the surface fish and Pachón analysed. Body length was used to correct for the heart measurements. (B) Uncorrected lateral ventricular area measurements show a similar pattern to the measurements corrected for body length, however variation between fish obscures the differences observed after correction. (C), While ventricular and atrial size are different between Pachón and surface fish, bulbus arteriosus size is not different. (D), correction for bulbus arteriosus area instead of body length shows similar results as for body length. (significance shown for Student's t-test. P<0.01 is denoted by **, P < 0.001 by ***, ns means not significant). A: atrium, V: Ventricle, OFT: outflow tract, scale bars: 100µm.

Supplementary Figure 2

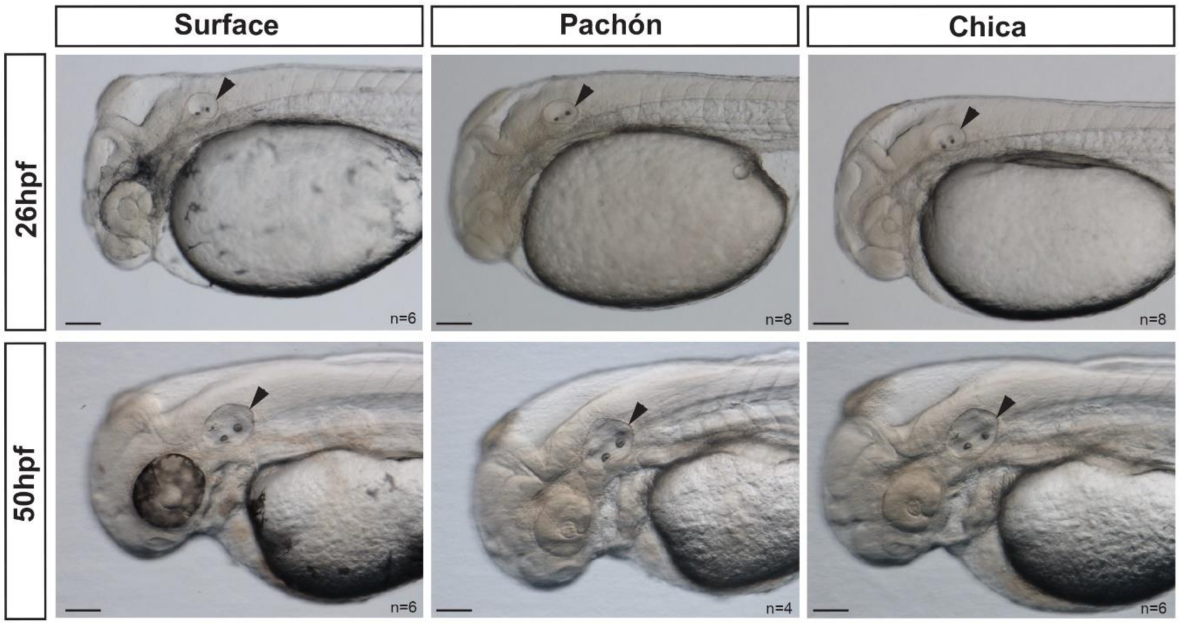

Supplementary Figure 2. Surface fish, Pachón and Chica embryos at 26hpf and 50hpf show similar otolith development, indicating that embryonic development occurs at similar speed between the populations and that the observed cardiac differences are not caused by developmental delay in cavefish populations compared to surface fish populations.
